# Synthesis, Characterization, and Anti-Inflammatory Activities of Methyl Salicylate Derivatives Bearing Piperazine Moiety

**DOI:** 10.3390/molecules21111544

**Published:** 2016-11-23

**Authors:** Jingfen Li, Yong Yin, Lisheng Wang, Pengyun Liang, Menghua Li, Xu Liu, Lichuan Wu, Hua Yang

**Affiliations:** 1 Department of Life Science, Huzhou Teachers’ College, Huzhou 313000, China; ljfljf@hutc.zj.cn; 2 School of Chemistry and Chemical Engineering, Guangxi University, Nanning 530004, China; yinyong1220@163.com (Y.Y.); w_lsheng@163.com (L.W.); gxulpyun@163.com (P.L.); lixiaomeng0108@163.com (M.L.); wendaoliuxu@163.com (X.L.)

**Keywords:** inflammation, salicylate, piperazine, derivatives, anti-inflammatory activity

## Abstract

In this study, a new series of 16 methyl salicylate derivatives bearing a piperazine moiety were synthesized and characterized. The in vivo anti-inflammatory activities of target compounds were investigated against xylol-induced ear edema and carrageenan-induced paw edema in mice. The results showed that all synthesized compounds exhibited potent anti-inflammatory activities. Especially, the anti-inflammatory activities of compounds **M15** and **M16** were higher than that of aspirin and even equal to that of indomethacin at the same dose. In addition, the in vitro cytotoxicity activities and anti-inflammatory activities of four target compounds were performed in RAW264.7 macrophages, and compound **M16** was found to significantly inhibit the release of lipopolysaccharide (LPS)-induced interleukin (IL)-6 and tumor necrosis factor (TNF)-α in a dose-dependent manner. In addition, compound **M16** was found to attenuate LPS induced cyclooxygenase (COX)-2 up-regulation. The current preliminary study may provide information for the development of new and safe anti-inflammatory agents.

## 1. Introduction

Inflammation is a complex biological process that occurs when body tissues are exposed to hazardous stimuli, such as irritants and pathogens [1]. Inflammation seriously threatens human health, and exaggerated and prolonged inflammation may cause various diseases, including arthritis, sepsis, and even cancer [2]. Presently, the most widely used drugs in inflammation treatment are non-steroidal anti-inflammatory drugs (NSAIDs), accounting for 35% of the global market of prescription of pain medications [3,4]. The common NSAIDs, such as aspirin and indomethacin, can inhibit cyclooxygenases (COX-1 and COX-2) [5,6], which are involved in the catalyzation of arachidonic acid to prostaglandins (PGs). Considering the significant toxicity of NSAIDs to the gastrointestinal tract and kidney [7], it is of great importance and urgent need to develop new anti-inflammation drugs [8].

Pharmaceuticals bearing a piperazine moiety are widely used in medicinal and pesticide chemistry [9]. Piperazine can effectively change the physicochemical properties of drugs and improve their pharmacokinetic properties and biological activity. Piperazine derivatives can act as histamine and serotonin receptor antagonists in the control of inflammation [10,11,12].

Salicylates are a class of chemicals with favorable anti-inflammatory effects. A large number of studies have indicated that methyl salicylate derivatives exhibited great anti-inflammatory effects [13,14,15]. In the current study, methyl salicylate and piperazine were combined to synthesize a new series of methyl salicylate derivatives, and their anti-inflammation activities were screened in vitro and in vivo.

## 2. Results

### 2.1. Synthesis of Salicylate Derivatives Bearing Piperazine Moiety

**Scheme 1:**
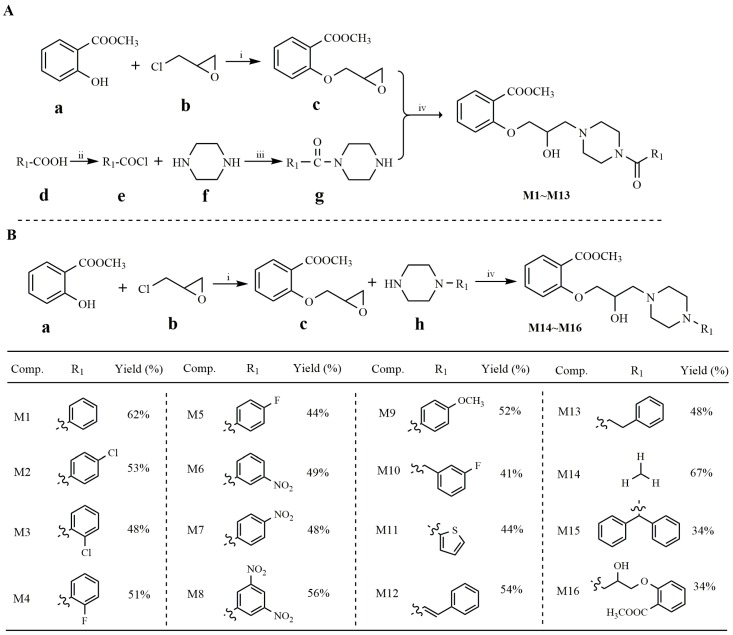
Synthetic routes of methyl salicylate derivatives. (**A**) Synthetic routes of compounds **M1**~**M13**. (**B**) Synthetic routes of compounds **M14**~**M16**. *Reagents and conditions*: (i) K_2_CO_3_, acetone, reflux, 30 h, yield = 85%; (ii) SOCl_2_, reflux, 2 h, yield = 71%–87%; (iii) Piperazine, AcOH, r.t., 3 h, yield = 64%–79%; (iv) toluene, reflux, 12 h, yield = 34%–67%.

The synthesis of target compounds is outlined in Scheme 1. Methyl salicylate (a) firstly reacted with 3-chloro-1,2-epoxypropane (b) to generate methyl-2-(oxiran-2-ylmethoxy) benzoate (c) (Scheme 1A, upper). Meanwhile, various aryl carboxylic acids (d) reacted with thionyl chloride to obtain the intermediate (e) which subsequently reacted with anhydrous piperazine (f) in the presence of acetic acid at room temperature, generating intermediate (g) (Scheme 1A, bottom). Then, based on combination principles, intermediate c reacted with g to generate products **M1**–**M13** in the presence of toluene, with yields ranging from 41% to 62% (Scheme 1A). For products **M14**–**M16**, raw material (h) reacted with methyl-2-(oxiran-2-ylmethoxy) benzoate (c) in the presence of toluene to generate the target methyl salicylate derivatives (**M14**–**M16**) (Scheme 1B), with yields ranging from 34% to 67%. All compounds were characterized by microanalytical and spectral data.

### 2.2. In Vivo Anti-Inflammatory Activities of Salicylate Derivatives Bearing Piperazine Moiety

**Figure 1:**
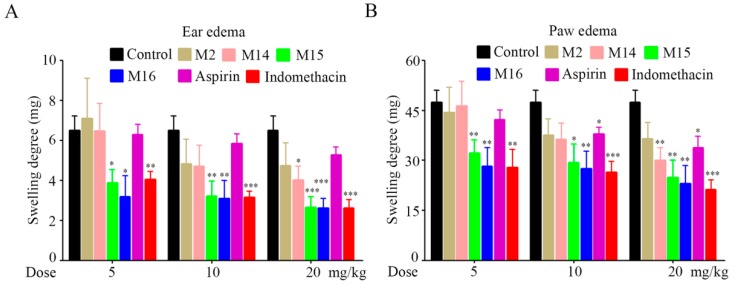
Target compounds showed dose-dependent inhibition on (**A**) ear and (**B**) paw edema in vivo. * *p* < 0.05, ** *p* < 0.01, *** *p* < 0.001 significantly different from the control value.

The in vivo anti-inflammatory activities of target compounds were evaluated against xylol-induced ear edema and carrageenan-induced paw edema in mice. All of the target compounds were administered at a dose of 100 mg/kg, and aspirin (100 mg/kg) and indomethacin (5 mg/kg) were used as standard controls. The results indicated that all compounds exhibited favorable anti-inflammatory activity compared to aspirin, except for compound **M10** (Table 1). Among the tested compounds, **M2**, **M14**, **M15**, and **M16** were much more potent than others, and compound **M16** exhibited the strongest anti-inflammatory activity. To further depict the dose-effect relationships of the four compounds (**M2**, **M14**, **M15**, and **M16**), various doses (5, 10, and 20 mg/kg) of the four compounds, aspirin, and indomethacin were intragastrically administered. The results showed that the anti-inflammatory activities of the four compounds were increased in a dose-dependent manner (Figure 1). Especially, the anti-inflammatory activities of compounds **M15** and **M16** were better than that of aspirin, and even equal to that of indomethacin at the same dose.

Piperazine-derived drugs are considered to be designer drugs which can be divided into two classes: the benzylpiperazines and the phenylpiperazines [16]. Although piperazine-derived drugs have been considered to be safe [16], adverse effects, such as dizziness, headache, hypersensitivity reactions, vomiting, and nausea have been reported from several experimental, clinical, and epidemiological studies [17,18]. Presently, the structure-side-effect relationships of drugs bearing a piperazine moiety with the central nervous system (CNS) have been revealed, indicating that drugs with the least CNS toxicity would be predicted to be those with low γ-aminobutyric acid (GABA)-binding ability and low overall lipophilicity [19]. Considering the potential toxicity of piperazine drugs, it is of great importance to detect their metabolites in humans or animals. Metabolism studies of piperazine designer drugs show that piperazine-derived drugs are mainly metabolized in the liver. The main metabolites are *N*-(4-hydroxy-3-methoxybenzyl)piperazine for the benzylpiperazines class, and hydroxyl-3-chloroaniline for phenylpiperazines, which finally turned into glucuronides and/or sulfates [16]. Our in vivo study showed that the oral dose of 100 mg/kg did not cause any gross behavioral alterations such as dizziness, respiratory distress, or mortality, indicating that the lethal dose of the target compounds is above 100 mg/kg body weight. Certainly, for future follow ups, we should carry out studies on acute toxicity, pharmacokinetics, and metabolism, especially for compound **M16**.

### 2.3. Cytotoxicity Assays of Compounds ***M2**, **M14**, **M15**,* and ***M16***

**Figure 2:**
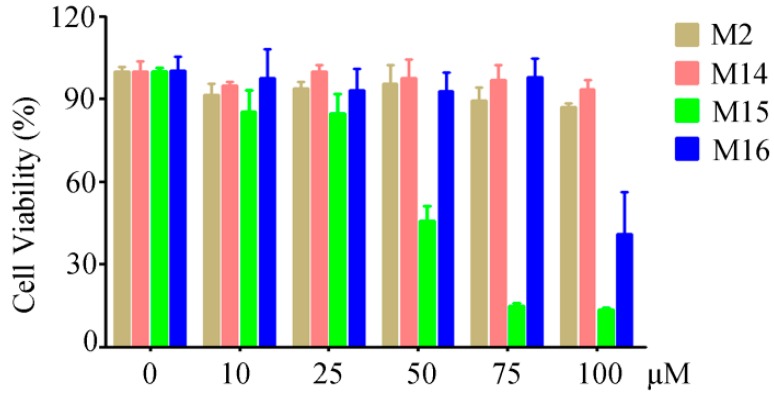
Cytotoxicity activities of **M2**, **M14**, **M15**, and **M16** against RAW264.7 macrophages.

To reveal the cytotoxicity of **M2**, **M14**, **M15**, and **M16**, a MTT (3-(4,5-Dimethylthiazol-2-yl)-2,5-diphenyltetrazolium bromide) assay was conducted in mouse RAW264.7 macrophages. The results indicated that compounds **M2**, **M14**, and **M16** showed no significant cytotoxicity with concentrations less than 75 µM (Figure 2). Compound **M15** displayed no significant cytotoxicity with concentrations less than 25 µM, but showed a great cytotoxicity at 50 µM (Figure 2). Thus, we used 6.25, 12.5, and 25 µM in the following ELISA (enzyme-linked immunoadsorbent assay) experiments.

### 2.4. Inhibitory Screening against Lipopolysaccharide (LPS)-Induced Interleukin (IL)-6 and Tumor Necrosis Factor (TNF)-α Release

**Figure 3:**
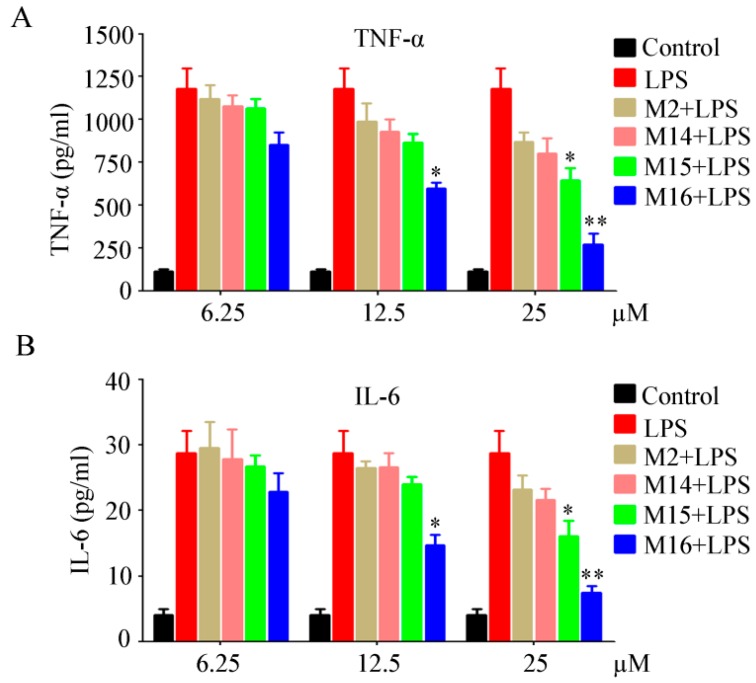
Target compounds inhibited lipopolysaccharide (LPS)-induced (**A**) tumor necrosis factor (TNF)-α and (**B**) interleukin (IL)-6 release in a dose-dependent manner in vitro. * *p* < 0.05, ** *p* < 0.01 significantly different from the LPS value.

TNF-α and IL-6, which are important pro-inflammatory mediators, can be triggered by lipopolysaccharide (LPS) [20,21]. TNF-α is released in macrophages upon LPS treatment. The secreted TNF-α or LPS subsequently induces the cells to release IL-6 [22]. In this study, the in vitro anti-inflammatory effects of four target compounds were evaluated in the macrophage RAW264.7 cells by measuring the expressions of TNF-α and IL-6 through ELISA assays. RAW264.7 macrophages were pretreated with different doses (6.25, 12.5, and 25 µM) for 2 h and then exposed to LPS (1 µg/mL) for an additional 22 h. The levels of TNF-α and IL-6 in the media were determined. The results displayed that all four target compounds could inhibit the release of TNF-α and IL-6. Especially, compound **M16** significantly inhibited TNF-α and IL-6 release in a dose-dependent manner (Figure 3). Compound **M15** significantly inhibited TNF-α and IL-6 release at 25 µM. These findings suggested that compounds **M15** and **M16** exerted their anti-inflammatory effects by suppressing the production of pro-inflammatory factors, which might partly explain their in vivo anti-inflammatory activities.

### 2.5. Compound ***M16*** Attenuates LPS Induced Cyclooxygenase-2 (COX-2) Up-Regulation

**Figure 4:**
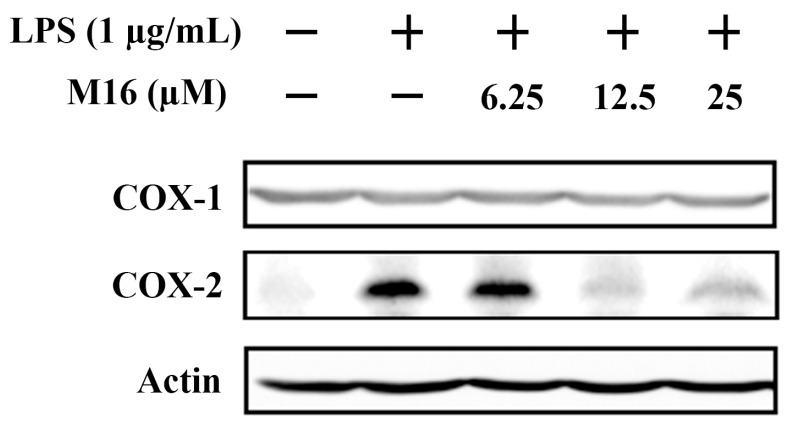
Compound **M16** attenuated LPS induced cyclooxygenase (COX)-2 up-regulation.

COX-2 is recognized as an inducible pro-inflammatory enzyme which is the rate-limiting enzyme of prostanoids synthesis. Many NSAIDs exert their anti-inflammatory activities by inhibiting COX-2 activity [23]. To further elucidate the underlying anti-inflammation mechanism of compound **M16**, Western blot assays were performed to detect the expression of cyclooxygenase-1 (COX-1) and cyclooxygenase-2 (COX-2). The results showed that COX-2 was up-regulated in RAW264.7 macrophages upon LPS stimulation. Pretreatment with **M16** could attenuate LPS-induced COX-2 up-regulation (Figure 4). These results inferred that compound **M16** might exert its anti-inflammatory activities by down-regulating COX-2 expression and inhibiting the release of TNF-α and IL-6. 

## 3. Experimental Section

### 3.1. Synthetic Details of Intermediates and Target Compounds

For intermediate c, 20 mL methyl salicylate (0.15 mol) was dissolved in 250 mL acetone, and then 40 g anhydrous potassium carbonate (0.30 mol) was added into the solution. The solution was kept stirring at room temperature, and 100 mL 3-chloro-1,2-epoxypropane (1.26 mol) was added and stirred at reflux for 30 h at 60 °C. The excess solvent was removed in vacuo, and then the crude product c was dissolved in 200 mL toluene. Then, the solvent was sequentially washed with water (150 mL × 2), 5% sodium hydroxide (200 mL × 2), and water (150 mL × 2). Finally, the organic phase was dried over anhydrous sodium sulfate overnight and then evaporated under vacuum.

For intermediate g, 0.02 mol aryl carboxylic acid was dissolved in 20 mL thionyl chloride, and the solution was stirred at reflux for 4 h. Then, 20 mL anhydrous chloroform was added into the solution, and thionyl chloride was removed in vacuo under reduced pressure to give intermediate e. Subsequently, intermediate e was dissolved in 10 mL chloroform and added dropwise into anthalazine dissolved in acetic acid. The solution was kept stirring at room temperature for 3 h, and then was alkalized in an ice bath with 20% sodium hydroxide till the pH value reached 9~10. Finally, the organic phase was extracted with chloroform (50 mL × 5), dried over anhydrous sodium sulfate overnight, and then evaporated under vacuum. 

For products **M1**–**M13**, intermediates c and g were dissolved in 80 mL toluene with a material ratio (1:1.1, mol ratio). The solution was stirred at reflux for 10 h at 95 °C. Finally, the excess solvent was evaporated off, and the residues were purified by silica gel-column chromatography with acetic ether/petroleum ether (7:3, *v*:*v*) as eluent to afford the corresponding products **M1**–**M13**.

For **M14** and **M15**, monosubstituted alkyl piperazine and intermediate c were dissolved in 80 mL toluene with a material ratio (1:1.1, mol ratio). The solution was stirred at reflux for 10 h at 95 °C. Finally, the excess solvent was evaporated off and the residues were filtered by silica gel-column chromatography with acetic ether/petroleum ether (6:4, *v*:*v*) as eluent to afford the corresponding products **M14**–**M15**.

For **M16**, anhydrous piperazine and intermediate c were dissolved in 80 mL toluene with a material ratio (1:2.2, mol ratio). The solution was stirred at reflux for 10 h at 95 °C. Finally, the excess solvent was evaporated off and the residues were filtered by silica gel-column chromatography with acetic ether/petroleum ether (6:4, *v*:*v*) as eluent to afford the corresponding product **M16**.

Compounds characterization data:

*Methyl 2-(3-(4-benzoylpiperazin-1-yl)-2-hydroxypropoxy)benzoate* **M1**: yellow liquid. IR (KBr, ν cm^−1^): 3442 (ν_O-H_); 3064 (ν_Ar-H_); 2943, 1498, 1444 (ν_C-H_); 1721, 1628 (ν_C=O_); ^1^H-NMR (CD_3_Cl, 600 MHz, δ ppm): 2.42~2.67 (m, 6H, -CH_2_-), 3.44 (m, 2H, -CH_2_-), 3.80 (m, 2H, -NCH_2_-), 3.87 (s, 3H, -CH_3_), 4.02 (m, 2H, -OCH_2_-), 4.14 (m, 1H, -OH), 4.19 (m, 1H, -CH-), 7.00 (m, 2H, ArH), 7.39 (m, 5H, ArH), 7.45 (m, 1H, ArH), 7.81 (m, 1H, ArH). MS (ESI, *m*/*z*): 399.35 ([M + H]^+^). Elemental analysis: calcd. for C_22_H_26_N_2_O_5_: C, 66.32; H, 6.58; N, 7.03; Found: C, 66.38; H, 6.90; N, 6.56.

*Methyl 2-(3-(4-(4-chlorobenzoyl)piperazin-1-yl)-2-hydroxypropoxy)benzoate* **M2**: yellow liquid. IR (KBr, ν cm^−1^): 3431 (ν_O-H_); 3055 (ν_Ar-H_); 2943, 1498, 1445 (ν_C-H_); 1718 (ν_C=O_); ^1^H-NMR (CD_3_Cl, δ ppm): 2.57~2.68 (m, 6H, -CH_2_-), 3.44 (m, 2H, -CH_2_-), 3.79 (m, 2H, -NCH_2_-), 3.87 (s, 3H, -CH_3_), 4.02 (m, 2H, -OCH_2_-), 4.18~4.21 (m, 2H, -CH(OH)-), 7.01 (m, 2H, ArH), 7.34 (d, *J* = 8.4 Hz, 2H, ArH), 7.38 (d, *J* = 8.4 Hz, 2H, ArH),7.46 (m, 1H, ArH), 7.81 (t, *J* = 3.9 Hz, 1H, ArH). MS (ESI, *m*/*z*): 433.35 ([M + H]^+^). Elemental analysis: calcd. for C_22_H_25_ClN_2_O_5_: C, 61.04; H, 5.82; N, 6.47; Found: C, 61.08; H, 5.84; N, 6.49.

*Methyl 2-(3-(4-(2-chlorobenzoyl)piperazin-1-yl)-2-hydroxypropoxy)benzoate* **M3**: yellow liquid. IR (KBr, ν cm^−1^): 3439 (ν_O-H_); 3072 (ν_Ar-H_); 2934, 1486, 1453 (ν_C-H_); 1727, 1633 (ν_C=O_); ^1^H-NMR (CD_3_Cl, δ ppm): 2.57~2.63 (m, 6H, -CH_2_-), 3.18~3.32 (m, 2H, -CH_2_-), 3.71 (m, 2H, -CH_2_-), 3.85 (s, 3H, -CH_3_), 4.02 (d, *J* = 7.2 Hz, 2H, -CH_2_-), 4.12 (m, 1H, -OH), 4.16 (d, *J* = 2.4 Hz, 1H, -CH-), 6.98 (m, 2H, ArH), 7.26~7.32 (m, 3H, ArH), 7.37 (m, H, ArH), 7.44 (t, *J* = 7.8 Hz, 1H, ArH), 7.79 (m, 1H, ArH). MS (ESI, *m*/*z*): 433.29 ([M + H]^+^). Elemental analysis: calcd. for C_22_H_25_ClN_2_O_5_: C, 61.04; H, 5.82; N, 6.47; Found: C, 61.02; H, 5.88; N, 6.44.

*Methyl 2-(3-(4-(2-fluorobenzoyl)piperazin-1-yl)-2-hydroxypropoxy)benzoate* **M4**: yellow liquid. IR (KBr, ν cm^−1^): 3456 (ν_O-H_); 3080 (ν_Ar-H_); 2942, 1498, 1427 (ν_C-H_); 1735, 1642 (ν_C=O_); ^1^H-NMR (CD_3_Cl, δ ppm): 2.47~2.62 (m, 6H, -CH_2_-), 3.30 (m, 2H, -CH_2_-), 3.77 (m, 2H, -CH_2_-), 3.81 (s, 3H, -CH_3_), 3.98 (m, 1H, -OH), 4.11~4.15 (m, 3H, -CHCH_2_-), 6.95 (t, *J* = 7.5 Hz, 2H, ArH), 7.03 (t, *J* = 9 Hz, 1H, ArH), 7.15 (t, *J* = 7.5 Hz, 1H, ArH), 7.32 (m, 2H, ArH), 7.41 (t, *J* = 7.8 Hz, 1H, ArH), 7.52 (d, *J* = 7.8 Hz, 1H, ArH). MS (ESI, *m*/*z*): 417.33 ([M + H]^+^). Elemental analysis: calcd. for C_22_H_25_FN_2_O_5_: C, 63.45; H, 6.05; N, 6.73; Found: C, 63.48; H, 6.01; N, 6.6.

*Methyl 2-(3-(4-(4-fluorobenzoyl)piperazin-1-yl)-2-hydroxypropoxy)benzoate* **M5**: yellow liquid. IR (KBr, ν cm^−1^): 3439 (ν_O-H_); 3072 (ν_Ar-H_); 2942, 1506, 1452 (ν_C-H_); 1718, 1642 (ν_C=O_); ^1^H-NMR (CD_3_Cl, δ ppm): 2.50~2.70 (m, 6H, -CH_2_-), 3.41 (m, 2H, -CH_2_-), 3.75 (m, 2H, -CH_2_-), 3.84 (s, 3H, -CH_3_), 3.99 (m, 1H, -CH-), 4.07~4.14 (m, 2H, -CH_2_-), 4.17 (m, 1H, -OH), 6.97 (m, 2H, ArH), 7.05 (t, *J* = 8.4 Hz, 2H, ArH), 7.38 (m, 2H, ArH), 7.43 (t, *J* = 7.8 Hz, 1H, ArH), 7.78 (d, *J* = 7.8 Hz, 1H, ArH). MS (ESI, *m*/*z*): 417.26 ([M + H]^+^). Elemental analysis: calcd. for C_22_H_25_FN_2_O_5_: C, 63.45; H, 6.05; N, 6.73; Found: C, 63.51; H, 6.08; N, 6.78.

*Methyl 2-(2-hydroxy-3-(4-(3-nitrobenzoyl)piperazin-1-yl)propoxy)benzoate* **M6**: yellow liquid. IR (KBr, ν cm^−1^): 3448 (ν_O-H_); 3080 (ν_Ar-H_); 2925, 1532, 1452 (ν_C-H_); 1726, 1642 (ν_C=O_); ^1^H-NMR (CD_3_Cl, δ ppm): 2.54~2.70 (m, 6H, -CH_2_-), 3.42 (m, 2H, -CH_2_-), 3.81 (m, 2H, -CH_2_-), 3.85 (s, 3H, -CH_3_), 4.01 (t, *J* = 7.5 Hz, 1H, -CH-), 4.08 (d, *J* = 3.6 Hz, 1H, -OH), 4.14~4.19 (m, 2H, -CH_2_-), 6.98 (t, *J* = 9.33 Hz, 2H, ArH), 7.44 (t, *J* = 7.8 Hz, 1H, ArH), 7.59 (t, *J* = 7.8 Hz, 1H, ArH), 7.72 (d, *J* = 7.8 Hz, 1H, ArH), 7.79 (d, *J* = 7.8 Hz, 1H, ArH), 8.25 (d, *J* = 8.4 Hz, 2H, ArH). MS (ESI, *m*/*z*): 444.12 ([M + H]^+^). Elemental analysis: calcd. for C_22_H_25_N_3_O_7_: C, 59.59; H, 5.68; N, 9.48; Found: C, 59.62; H, 5.65; N, 9.52.

*Methyl 2-(2-hydroxy-3-(4-(4-nitrobenzoyl)piperazin-1-yl)propoxy)benzoate* **M7**: yellow liquid. IR (KBr, ν cm^−1^): 3439 (ν_O-H_); 3080 (ν_Ar-H_); 2942, 1524, 1444 (ν_C-H_); 1727, 1634 (ν_C=O_); ^1^H-NMR (CD_3_Cl, δ ppm): 2.58~2.74 (m, 6H, -CH_2_-), 3.38 (m, 2H, -CH_2_-), 3.81 (m, 2H, -CH_2_-), 3.86 (s, 3H,-CH_3_), 4.02 (m, 1H, -OH), 4.10~4.21 (m, 3H, -CHCH_2_-), 7.01 (m, 2H, ArH), 7.46 (m, 1H, ArH), 7.56 (m, 2H, ArH), 7.81 (m, 1H, ArH), 8.26 (m, 2H, ArH). MS (ESI, *m*/*z*): 444.14 ([M + H]^+^). Elemental analysis: calcd. for C_22_H_25_N_3_O_7_: C, 59.59; H, 5.68; N, 9.48; Found: C, 59.63; H, 5.64; N, 9.53.

*Methyl 2-(3-(4-(3,5-dinitrobenzoyl)piperazin-1-yl)-2-hydroxypropoxy)benzoate* **M8**: yellow liquid. IR (KBr, ν cm^−1^): 3456 (ν_O-H_); 3097 (ν_Ar-H_); 2926, 1548, 1445 (ν_C-H_); 1727, 1650 (ν_C=O_); ^1^H-NMR (CD_3_Cl, δ ppm): 2.61~2.78 (m, 6H, -CH_2_-), 3.45 (m, 2H, -CH_2_-), 3.84 (m, 2H, -CH_2_-), 3.87 (s, 3H, -CH_3_), 4.02~4.07 (m, 2H, -CH_2_-), 4.17 (s, 1H, -CH-), 4.22 (m, 1H, -OH), 7.02 (m, 2H ArH,), 7.26 (s, 1H, ArH), 7.48 (t, *J* = 7.8 Hz, 1H, ArH), 7.82 (d, *J* = 7.8 Hz, 1H, ArH), 8.60 (s, 2H, ArH), 9.09 (s, 1H, ArH). MS (ESI, *m*/*z*): 489.28 ([M + H]^+^). Elemental analysis: calcd. for C_22_H_24_N_4_O_9_: C, 54.10; H, 4.95; N, 11.47; Found: C, 54.12; H, 4.98; N, 11.44.

*Methyl 2-(2-hydroxy-3-(4-(4-methoxybenzoyl)piperazin-1-yl)propoxy)benzoate* **M9**: yellow liquid. IR (KBr, ν cm^−1^): 3431 (ν_O-H_); 3072 (ν_Ar-H_); 2942, 1515, 1446 (ν_C-H_); 1727, 1625 (ν_C=O_); ^1^H-NMR (CD_3_Cl, δ ppm): 2.48~2.70 (m, 8H, -CH_2_-), 3.79 (m, 3H, -CH_3_), 3.84 (s, 3H, CH_3_), 4.00 (m, 1H, -OH), 4.15 (m, 3H, -CHCH_2_-), 6.87 (d, *J* = 7.8 Hz, 2H, ArH), 6.97 (t, *J* = 8.1 Hz, 2H, ArH), 7.34 (d, *J* = 7.8 Hz, 2H, ArH), 7.43 (t, *J* = 7.8 Hz, 1H, ArH), 7.78 (d, *J* = 7.8 Hz, 1H, ArH). MS (ESI, *m*/*z*): 429.30 ([M + H]^+^). Elemental analysis: calcd. for C_23_H_28_N_2_O_6_: C, 64.47; H, 6.59; N, 6.54; Found: C, 64.43; H, 6.54; N, 6.58.

*Methyl 2-(3-(4-(2-(3-fluorophenyl)acetyl)piperazin-1-yl)-2-hydroxypropoxy)benzoate* **M10**: yellow liquid. IR (KBr, ν cm^−1^): 3448 (ν_O-H_); 3074 (ν_Ar-H_); 2951, 1486, 1445 (ν_C-H_); 1718, 1642 (ν_C=O_); ^1^H-NMR (CD_3_Cl, δ ppm): 2.36~2.61 (m, 6H, -CH_2_-), 3.42 (t, *J* = 5.1 Hz, 2H, -CH_2_-), 3.58~3.70 (m, 5H, -CHCH_2_-,-CH_2_-), 3.83 (s, 3H, -CH_3_), 3.98 (m, 1H, -OH), 4.08~4.15 (m, 2H, -CH_2_-), 6.89~6.98 (m, 5H, ArH), 7.24 (d, *J* = 6.0 Hz, 1H, ArH), 7.42 (m, 1H, ArH), 7.78 (t, *J* = 3.9 Hz, 1H, ArH). MS (ESI, *m*/*z*): 431.40 ([M + H]^+^). Elemental analysis: calcd. for C_23_H_27_FN_2_O_5_: C, 64.17; H, 6.32; N, 6.51; Found: C, 64.15; H, 6.38; N, 6.48.

*Methyl 2-(2-hydroxy-3-(4-(thiophene-2-carbonyl)piperazin-1-yl)propoxy)benzoate* **M11**: yellow liquid. IR (KBr, ν cm^−1^): 3447 (ν_O-H_); 3089 (ν_Ar-H_); 2934, 1523, 1452 (ν_C-H_); 1727, 1625 (ν_C=O_); ^1^H-NMR (CD_3_Cl, δ ppm): 2.53~2.64 (m, 6H, -CH_2_-), 3.68~3.80 (m, 4H, -CH_2_-), 3.83 (s, 3H, -CH_3_), 4.00 (m, 1H, -OH), 4.05~4.18 (m, 3H, -CHCH_2_-), 6.98 (m, 3H, ArH), 7.24 (m, 1H, ArH), 7.40 (m, 2H, ArH), 7.77 (m, 1H, ArH). MS (ESI, *m*/*z*): 405.16 ([M + H]^+^). Elemental analysis: calcd. for C_20_H_24_N_2_O_5_S: C, 59.39; H, 5.98; N, 6.93; Found: C, 59.44; H, 6.02; N, 6.89.

*Methyl 2-(3-(4-cinnamoylpiperazin-1-yl)-2-hydroxypropoxy)benzoate* **M12**: yellow liquid. IR (KBr, ν cm^−1^): 3448 (ν_O-H_); 3097 (ν_Ar-H_); 2934, 1527, 1453 (ν_C-H_); 1718, 1642 (ν_C=O_); ^1^H-NMR (CD_3_Cl, δ ppm): 2.50~2.68 (m, 6H, -CH_2_-), 3.50~3.78 (m, 4H, -CH_2_-), 3.86 (s, 3H, -CH_3_), 4.01~4.12 (m, 2H, -CH(OH)-), 4.15~4.21 (m, 2H, -CH_2_-), 6.85 (d, *J* = 15.6 Hz, 1H, ArH), 6.97~7.00 (t, *J* = 7.2 Hz, 2H, -CH = CH-), 7.31~7.36 (m, 3H, ArH), 7.44 (t, *J* = 7.8 Hz, 1H, ArH), 7.49 (d, *J* = 7.2 Hz, 2H, ArH), 7.64 (d, *J* = 15 Hz, 1H, ArH), 7.80 (d, *J* = 7.8 Hz, H, ArH). MS (ESI, *m*/*z*): 425.31 ([M + H]^+^). Elemental analysis: calcd. for C_24_H_28_N_2_O_5_: C, 67.91; H, 6.65; N, 6.60; Found: C, 67.88; H, 6.64; N, 6.64.

*Methyl 2-(2-hydroxy-3-(4-(3-phenylpropanoyl)piperazin-1-yl)propoxy)benzoate* **M13**: yellow liquid. IR (KBr, ν cm^−1^): 3456 (ν_O-H_); 3038 (ν_Ar-H_); 2951, 1498, 1452 (ν_C-H_); 1718, 1642 (ν_C=O_); ^1^H-NMR (CD_3_Cl, δ ppm): 2.41~2.61 (m, 8H, -CH_2_-), 2.95 (t, *J* = 7.8 Hz, 2H, -CH_2_-), 3.8 (t, *J* = 5.1 Hz, 2H, -CH_2_-), 3.63 (m, 2H, -CH_2_-), 3.86 (s, 3H, -CH_3_), 4.01 (m, 1H, -OH), 4.10~4.19 (m, 3H, -CHCH_2_-), 6.98 (m, 2H, ArH), 7.19 (t, *J* = 8.7 Hz, 3H, ArH), 7.27 (t, *J* = 7.5 Hz, 2H, ArH), 7.45 (m, 1H, ArH), 7.80 (d, *J* = 7.2 Hz, 1H, ArH). MS (ESI, *m*/*z*): 427.34 ([M + H]^+^). Elemental analysis: calcd. for C_24_H_30_N_2_O_5_: C, 67.59; H, 7.09; N, 6.57; Found: C, 67.56; H, 7.06; N, 6.61.

*Methyl 2-(2-hydroxy-3-(4-methylpiperazin-1-yl)propoxy)benzoate* **M14**: yellow liquid. IR (KBr, ν cm^−1^): 3439 (ν_O-H_); 3072 (ν_Ar-H_); 2951, 1486, 1453 (ν_C-H_); 1718, 1608 (ν_C=O_); ^1^H-NMR (CD_3_Cl, δ ppm): 2.26 (s, 3H, -CH_3_), 2.30~2.70 (m, 10H, -CH_2_-), 3.85 (s, 3H, -CH_3_), 4.00 (m, 1H, -OH), 4.10~4.14 (m, 3H, -CHCH_2_-), 6.97 (m, 2H, ArH), 7.43 (t, *J* = 7.8 Hz, 1H, ArH), 7.79 (m, 1H, ArH). MS (ESI, *m*/*z*): 309.20 ([M + H]^+^). Elemental analysis: calcd. for C_16_H_24_N_2_O_4_: C, 62.32; H, 7.84; N, 9.08; Found: C, 62.29; H, 7.88; N, 9.04.

*Methyl 2-(3-(4-benzhydrylpiperazin-1-yl)-2-hydroxypropoxy)benzoate* **M15**: yellow liquid. IR (KBr, ν cm^−1^): 3456 (ν_O-H_); 3114 (ν_Ar-H_); 2942, 1489, 1452 (ν_C-H_); 1718, 1642 (ν_C=O_); ^1^H-NMR (CD_3_Cl, δ ppm): 2.36~2.70 (m, 10H, -CH_2_-), 3.87 (s, 3H, -CH_3_), 3.93~4.08 (m, 2H, -CH(OH)-), 4.09~4.16 (m, 2H, -CH_2_-), 4.23 (s, 1H, -CH-), 6.99 (t, *J* = 7.5 Hz, 2H, ArH), 7.17 (t, *J* = 7.8 Hz, 2H, ArH), 7.27 (m, 4H, ArH), 7.40~7.46 (m, 5H, ArH), 7.81 (d, *J* = 7.8 Hz, 1H, ArH). MS (ESI, *m*/*z*): 416.43 ([M + H]^+^). Elemental analysis: calcd. for C_28_H_32_N_2_O_4_: C, 73.02; H, 7.00; N, 6.08; Found: C: 73.08; H, 7.03; N, 6.11.

*Dimethyl 2,2′-((piperazine-1,4-diylbis(2-hydroxypropane-3,1-diyl))bis(oxy))dibenzoate* **M16**: yellow liquid. IR (KBr, ν cm^−1^): 3430 (ν_O-H_); 3080 (ν_Ar-H_); 2934, 1506, 1453 (ν_C-H_); 1727, 1633 (ν_C=O_); ^1^H-NMR (CD_3_Cl, δ ppm): 2.54~2.72 (m, 2 × 6H, -CH_2_-), 3.85 (s, 2 × 3H, -CH_3_), 4.00 (t, *J* = 4.5 Hz, 2 × 1H, -OH), 4.13 (m, 2 × 2H, -CH_2_-), 6.97 (m, 2 × 2H, ArH), 7.42 (m, 2 × 1H, ArH), 7.79 (m, 2 × 1H, ArH). MS (ESI, *m*/*z*): 503.35 ([M + H]^+^). Elemental analysis: calcd. for C_26_H_34_N_2_O_8_: C, 62.14; H, 6.82; N, 5.57; Found: C, 62.18; H, 6.79; N, 5.54.

### 3.2. Cell Culture and Cytotoxicity Assays

Mouse RAW264.7 macrophages were purchased from American Type Culture Collection (ATCC) and cultured in DMEM (Dulbecco modified Eagle medium) supplemented with 10% fetal bovine serum (FBS) and 1% penicillin–streptomycin at 37 °C, in an atmosphere of 95% air and 5% CO_2_ under humidified conditions. For cytotoxicity assay, cells were seeded in 96-well plates at the density of 2 × 10^4^ cells per well and incubated for 16 h. The medium was removed, and the cells were then incubated with different concentrations of synthesized compounds for indicated times, followed by 2–3 h treatment with MTT. After MTT removal, 150 µL dimethyl sulfoxide (DMSO) was added into each well and absorption values was measured at 490 nm using a SpectraMAX M5 plate reader (Molecular Devices Inc. San Francisco, CA, USA). Three independent experiments were conducted, and samples were analyzed in triplicate for each experiment.

### 3.3. In Vivo Anti-Inflammatory Activity Assays in Ear and Paw Edema

The animal studies were approved by the Institutional Review Board of Guangxi Medical University. All animal studies were conducted according to protocols approved by the Animal Ethics Committee of Guangxi Medical University (Nanning, China). The anti-inflammatory activity was evaluated on xylene-induced ear edema and carrageenan-induced mice paw edema [24]. Briefly, the mice (20 ± 2 g, 10/group) were fasted with free access to water for at least 16 h, and then randomly divided into two groups: the dosage and control groups. Ear edema and paw edema was induced by applying xylene on the left ear and subcutaneous injection of carrageenan in the left toe of each mouse, respectively. One hour before edema induction, the mice were intragastrically administered with target drugs in 1% carboxymethyl cellulose solution or control solvent. Three hours later, the mice were euthanized by cervical dislocation, and right ears or paws were cut and weighed for swelling degree and swelling inhibition calculation.

### 3.4. ELISA Assay

Mouse RAW264.7 macrophages were pretreated with compounds (**M2**, **M14**, **M15**, and **M16**) or vehicle control for 2 h, then treated with LPS (1 µg/mL) for 22 h. After treatment, the cells and media were collected separately. The levels of IL-6 and TNF-α in the media were determined using ELISA kit (eBioscience, San Diego, CA, USA). The total protein was extracted from the collected cells and determined using Bio-Rad protein assay. The amount of IL-6 and TNF-α was normalized to the total protein amount.

### 3.5. Western Blot Assays

For Western blot assay, cells were lysed in RIPA (Radio-Immunoprecipitation Assay) buffer supplemented with protease inhibitors. Proteins (20 μg) were subjected to 6%–15% SDS-PAGE, electrophoresed, and transferred on to a nitrocellulose membrane. After blocking with 5% non-fat milk in *tris*-buffered saline, the membrane was washed and incubated with the indicated primary and secondary antibodies and detected using the Luminescent Image Analyser LSA 4000 (GE, Fairfield, CO, USA).

### 3.6. Statistical Analysis

The data are presented as the mean ± SD. Differences between data groups were evaluated for significance using Student’s *t*-test of unpaired data. *p* values < 0.05 were considered statistically significant.

## 4. Conclusions

In summary, a new series of methyl salicylate derivatives were synthesized and characterized. The in vivo anti-inflammatory results revealed that all target compounds exhibited potent anti-inflammatory activity compared to aspirin, except compound **M10**. In addition, compounds **M2**, **M14**, **M15**, and **M16** exhibited strong anti-inflammatory activity in a dose-dependent manner in vivo. Especially, the anti-inflammatory activities of compounds **M15** and **M16** were higher than that of aspirin, and even equal to that of indomethacin at the same dose. Furthermore, compound **M16** could significantly inhibit LPS-induced IL-6 and TNF-α release and down-regulate the expression of COX-2 in a dose-dependent manner. Our findings may provide information on potentially new and safe anti-inflammatory agents for further studies.

## Figures and Tables

**Table 1:** Anti-inflammatory (in vivo) activity of the target compounds against xylol-induced ear edema and carrageenan-induced paw edema in mice.

Compound	Dose (mg/kg)	Ear Edema		Paw Edema		Dose (mmol/kg)
		Swelling Degree (mg)	Inhibition (%)	Swelling Degree (mg)	Inhibition (%)	
Control		8.36 ± 2.50	-	94.93 ± 8.46	-	
**M1**	100	2.82 ± 0.56 ***	66.25	59.31 ± 6.25 ***	37.52	0.25
**M2**	100	2.29 ± 0.47 ***	72.65	43.44 ± 8.65 ***	54.24	0.23
**M3**	100	2.42 ± 0.44 ***	71.09	50.58 ± 7.40 ***	46.72	0.23
**M4**	100	2.70 ± 0.49 ***	67.67	54.82 ± 5.03 **	42.25	0.24
**M5**	100	4.61 ± 1.11 **	44.82	57.98 ± 4.94 **	38.92	0.24
**M6**	100	4.54 ± 1.16 **	45.70	61.04 ± 5.58 ***	35.70	0.23
**M7**	100	5.03 ± 1.77 *	39.80	64.58 ± 6.75 ***	31.97	0.23
**M8**	100	4.05 ± 1.55 **	51.55	59.60 ± 6.86 ***	37.21	0.20
**M9**	100	4.63 ± 1.43 **	44.65	59.22 ± 5.21 ***	37.62	0.23
**M10**	100	4.07 ± 1.08 **	51.30	79.22 ± 7.06 **	16.55	0.23
**M11**	100	3.74 ± 1.13 ***	55.31	49.24 ± 7.79 ***	48.13	0.25
**M12**	100	4.31 ± 1.37 **	48.40	59.91 ± 7.72 ***	36.89	0.24
**M13**	100	3.61 ± 1.17 ***	56.80	53.43 ± 6.01 ***	43.72	0.23
**M14**	100	2.43 ± 0.75 ***	70.97	43.64 ± 6.37 ***	54.03	0.32
**M15**	100	2.13 ± 1.09 ***	74.48	41.42 ± 7.07 ***	56.37	0.24
**M16**	100	1.53 ± 0.55 ***	81.73	33.43 ± 6.92 ***	64.78	0.20
Aspirin	100	4.85 ± 1.70 *	41.96	67.24 ± 8.68 ***	29.16	0.56
Indomethacin	5	4.96 ± 1.09 **	40.66	64.13 ± 8.91 ***	32.45	0.01

* *p* < 0.05, ** *p* < 0.01, *** *p* < 0.001 significantly different from the control value.
